# Supplementary material for: Synergistic recruitment of UbcH7~Ub and phosphorylated Ubl domain triggers parkin activation
Source: EMBO J. 2018 Nov 16;37(23):e100014. doi: 10.15252/embj.2018100014 (PMC6276879; doi:10.15252/embj.2018100014)
Supplement: Supplementary file 1 — Appendix [file EMBJ-37-e100014-s001.pdf]

Appendix

Synergistic Recruitment of UbcH7~Ub and Phosphorylated Ubl Domain Triggers Parkin Activation

Tara E.C. Condos, Karen M. Dunkerley, E. Aisha Freeman, Kathryn R. Barber, Jacob D. Aguirre, Viduth K. Chaugule, Yiming Xiao, Lars Konermann, Helen Walden and Gary S. Shaw

Table of Contents

Figure S1. Selected deuterium uptake curves measured from HDX experiments using mass spectrometry

Figure S2. Parkin peptide coverage map

Figure S3. UbcH7-Ub binding interface with R0RBR:pUb

Figure S4. The closed UbcH7-Ub state is released upon binding to R0RBR:pUb

Figure S5. Effect of W403A substitution in R0RBR parkin

Figure S6. Selected deuterium uptake curves measured from HDX experiments using mass spectrometry

Figure S7. Reaction of the catalytic C431 residue in parkin, parkin^{W403A} and the RING2(Rcat) with a UbVS probe

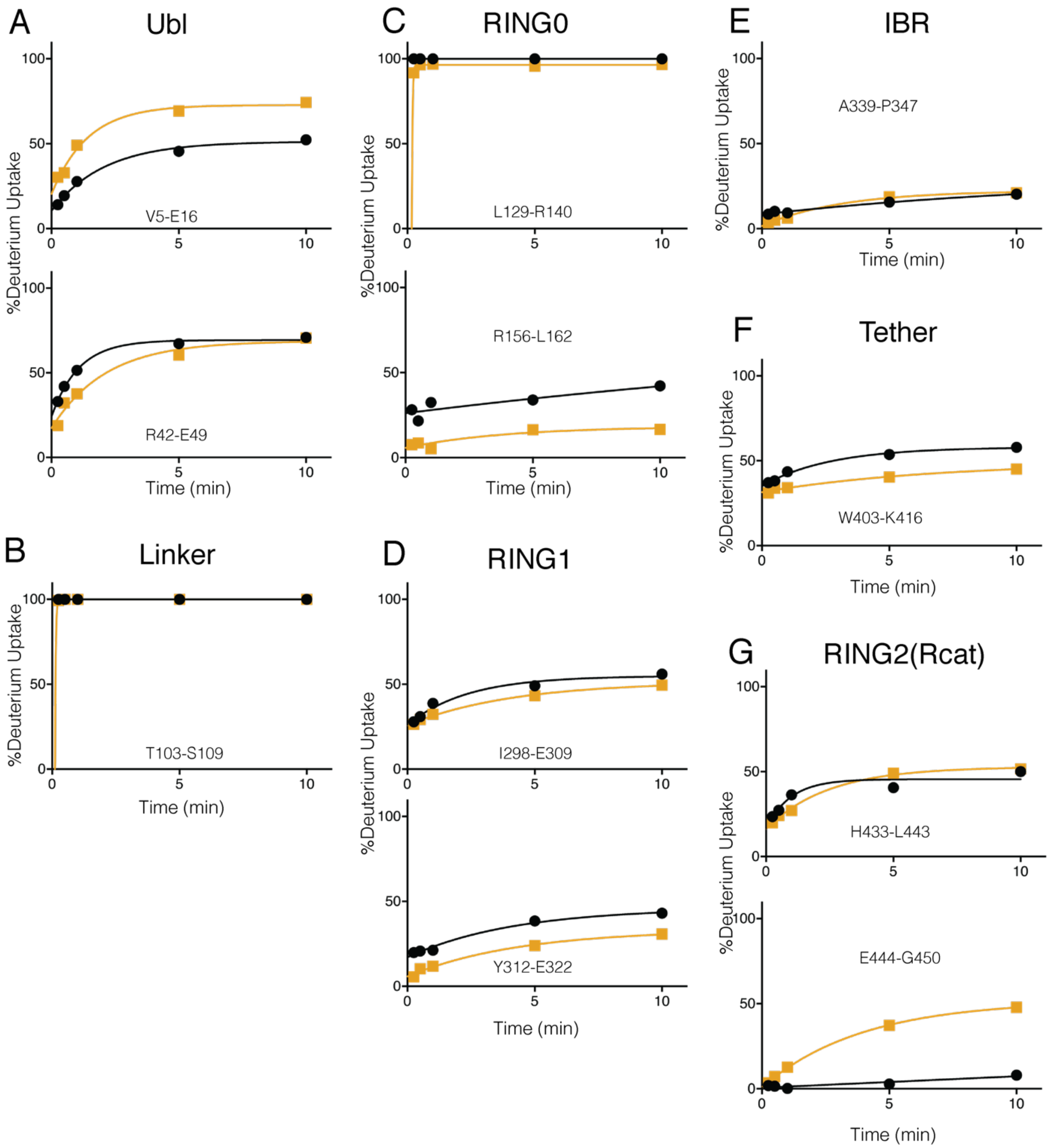

Figure S1. Selected deuterium uptake curves measured from HDX experiments using mass spectrometry. The curves show the % deuterium incorporation for a given peptide as a function of time (minutes) for parkin (●) and pParkin:pUb (■) in (A) Ubl/pUbl, (B) linker, (C) RING0, (D) RING1, (E) IBR, (F) tether and (G) RING2/Rcat domains.

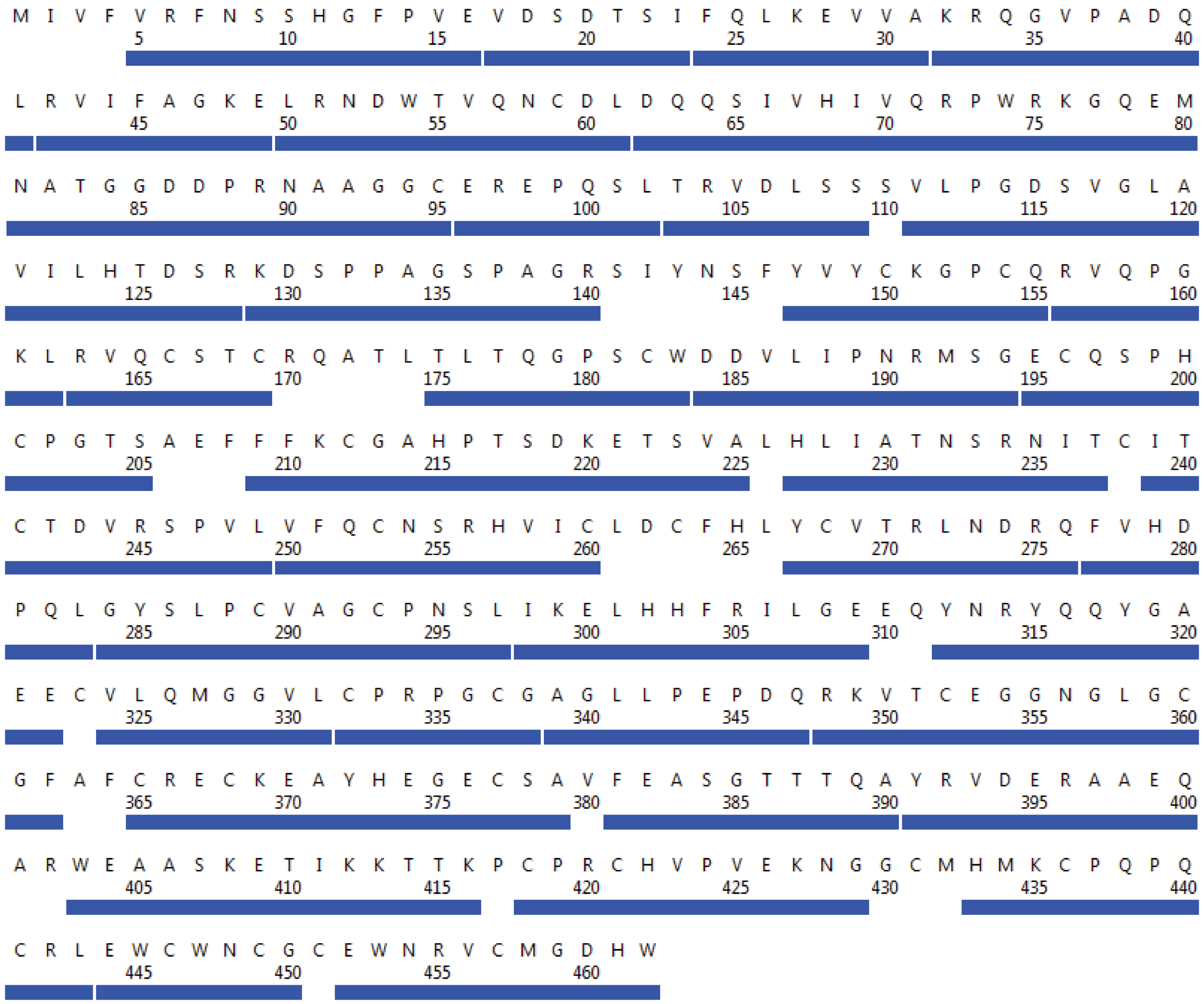

Figure S2. Parkin peptide coverage map. The parkin peptide map, which shows the subset of contiguous peptides of parkin used for all MS-HDX experiments. This consistent group of peptides was used for all measurements involving parkin, the pParkin:pUb complex and the pParkin:pUb-UbcH7-Ub complex. Coverage is indicated at 91.3% coverage.

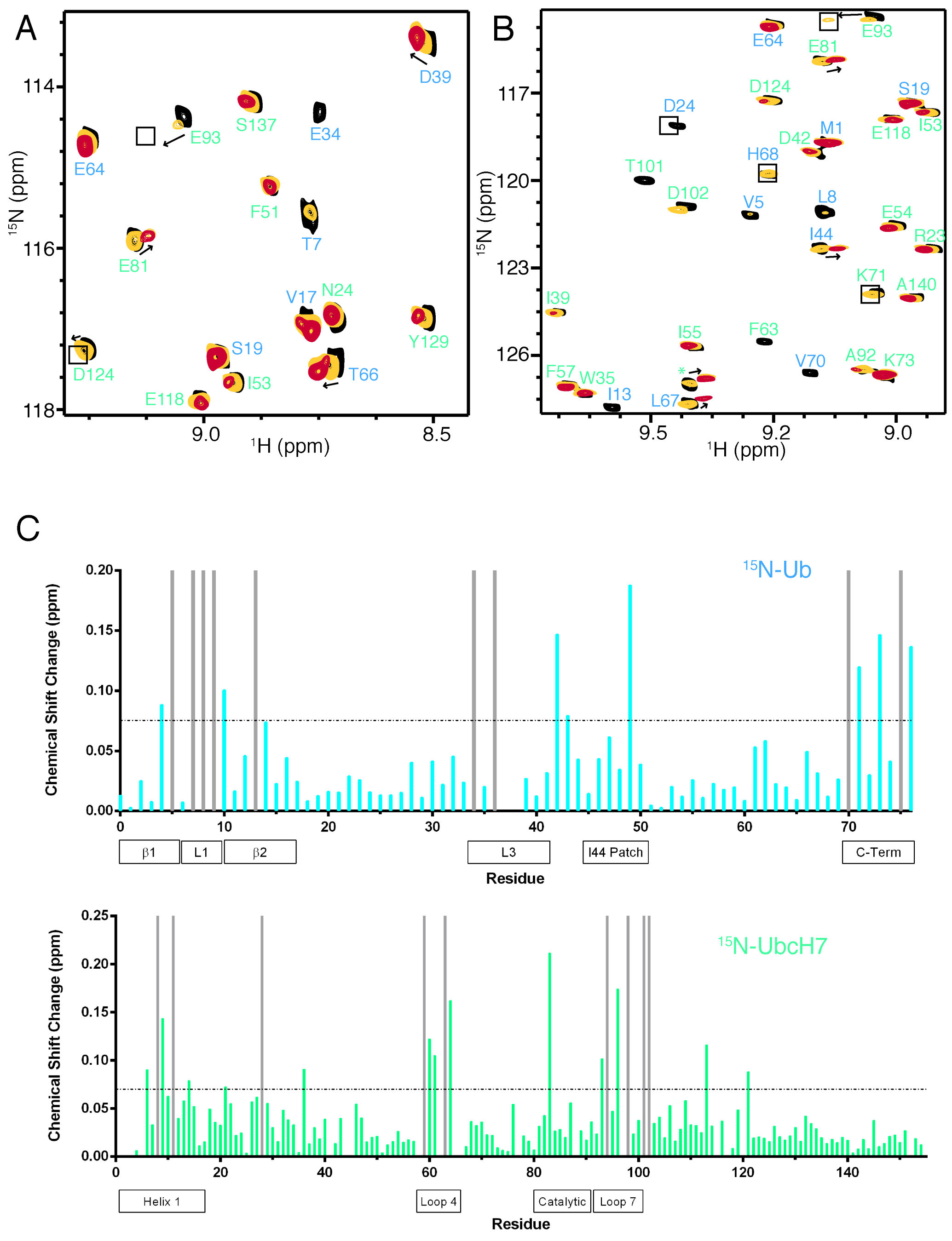

Figure S3. UbcH7-Ub binding interface with R0RBR:pUb. (A, B) Selected regions from ^{1}H-^{15}N TROSY NMR spectra of ^{2}H,^{12}C,^{15}N-labelled UbcH7-Ub in the absence (black contours) and presence of 0.5 equivalents (yellow contours) or 1 equivalent (red contours) ^{2}H-labelled R0RBR:pUb. The spectra are labelled for UbcH7 (green) or Ub (blue) in the UbcH7-Ub conjugate. (C) Measured chemical shift changes in either UbcH7 or Ub in the UbcH7-Ub conjugate upon R0RBR:pUb binding at 1 equivalent R0RBR:pUb. Grey bars indicate that the resonance was undetectable. The horizontal dashed line indicates the average CSP +1 standard deviation. Data was collected in 25 mM HEPES, 50 mM NaCl, 500 μM TCEP, pH 7.0 buffer at 25°C.

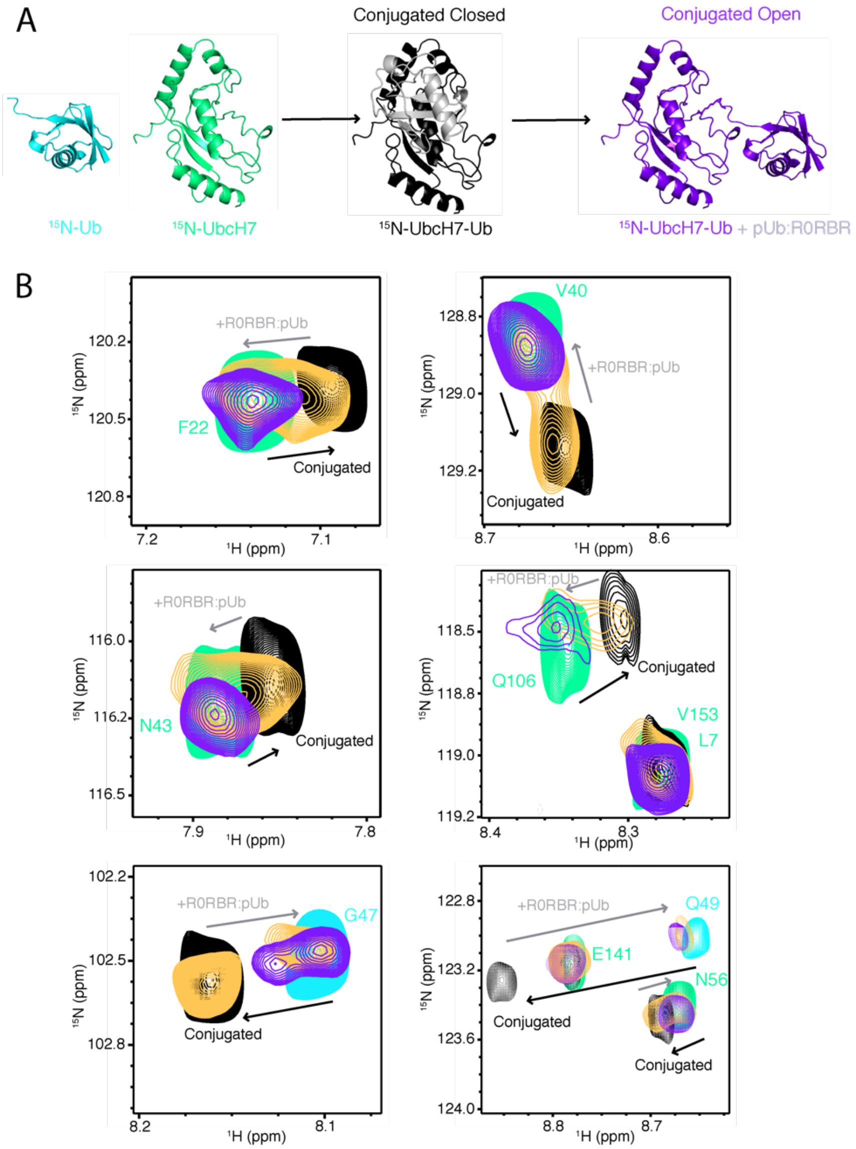

Figure S4. The closed UbcH7-Ub state is released upon binding to R0RBR:pUb. (A) Ribbon diagram representations of UbcH7 and Ub and the "closed" and "open" UbcH7-Ub states formed upon conjugation. (B) ^{1}H-^{15}N TROSY NMR spectra of unconjugated ^{2}H,^{12}C,^{15}N-labelled UbcH7 (green contours, top four panels) or ^{2}H,^{12}C,^{15}N-labelled Ub (cyan contours, bottom two panels) along with corresponding residue assignments. These two labelled moieties were then conjugated to form the UbcH7-Ub product (black contours). UbcH7 resonances for F22, V40, N43 and Q106 and Ub resonances for G47 and Q49 are perturbed upon conjugate formation representative of the "closed" UbcH7-Ub state. ^{2}H,^{12}C,^{15}N-labelled UbcH7-Ub was titrated with 0.5 equivalents (yellow contours) and 1 equivalent (purple contours) of ^{2}H-labelled R0RBR:pUb. In all cases the UbcH7 and Ub resonances revert to a position near that for the unconjugated forms of UbcH7 and Ub indicative of "open" conformation. All resonances exhibit slow or slow-intermediate exchange indicating the UbcH7-Ub conjugate interacts tightly R0RBR:pUb.

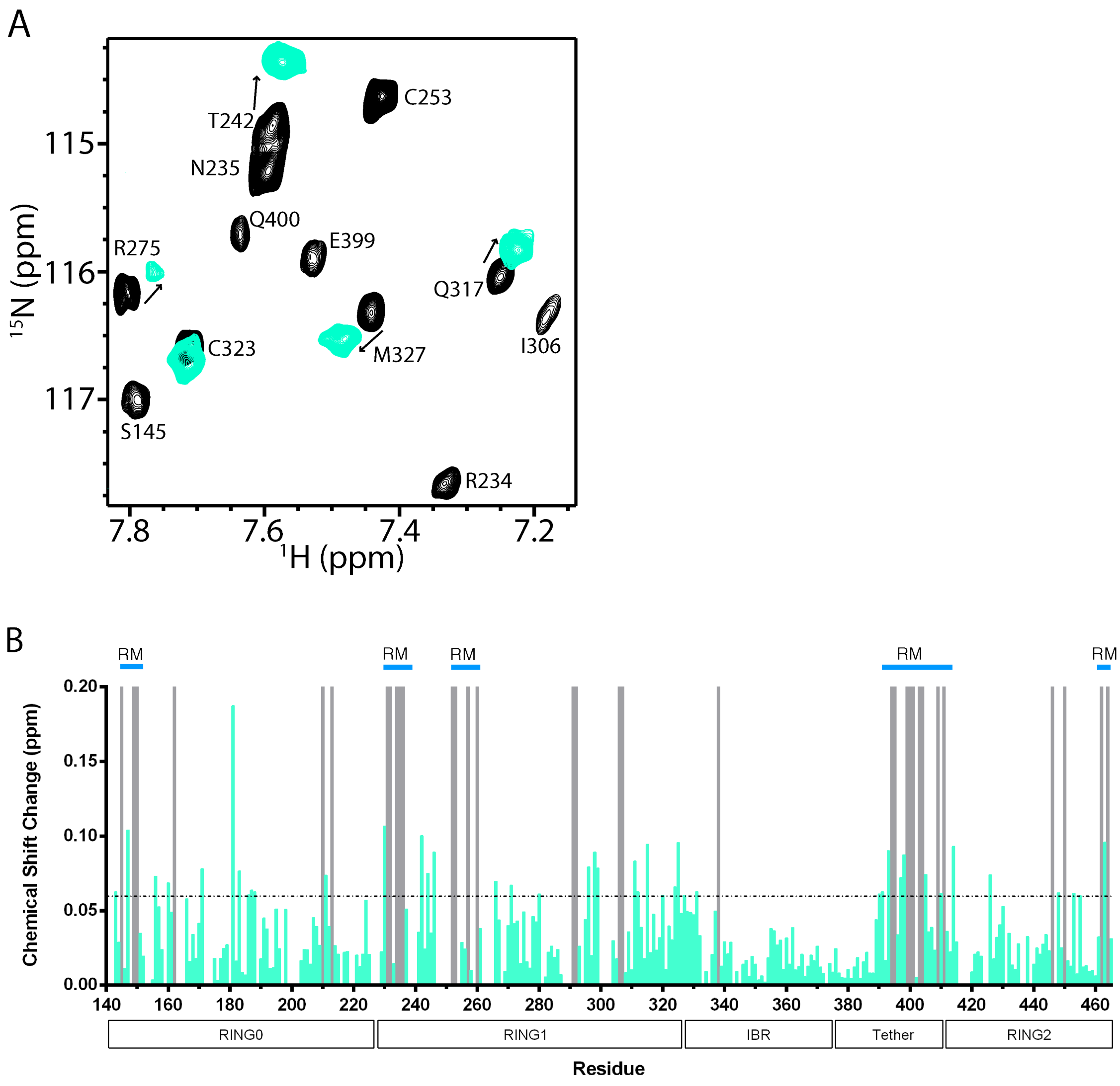

Figure S5. Effect of W403A substitution in R0RBR parkin. (A) Selected region of ^{1}H-^{15}N TROSY NMR spectra for ^{15}N-labelled R0RBR parkin (black contours) and R0RBR^{W403A} parkin (aquamarine contours) are shown labelled with assignments from R0RBR parkin. The spectra show that several signals for R0RBR^{W403A} are shifted due to a change in environment from the W403A substitution. (B) The chemical shift changes between R0RBR parkin and R0RBR^{W403A} parkin were calculated and plotted. Grey bars indicate that the resonance in R0RBR^{W403A} parkin was shifted significantly and could not be assigned. Above the plot regions identified from UbcH7-Ub binding experiments to the R0RBR:pUb complex due to remodelling of the RING0/RING1/RING2 interface are indicated (RM, blue bars).

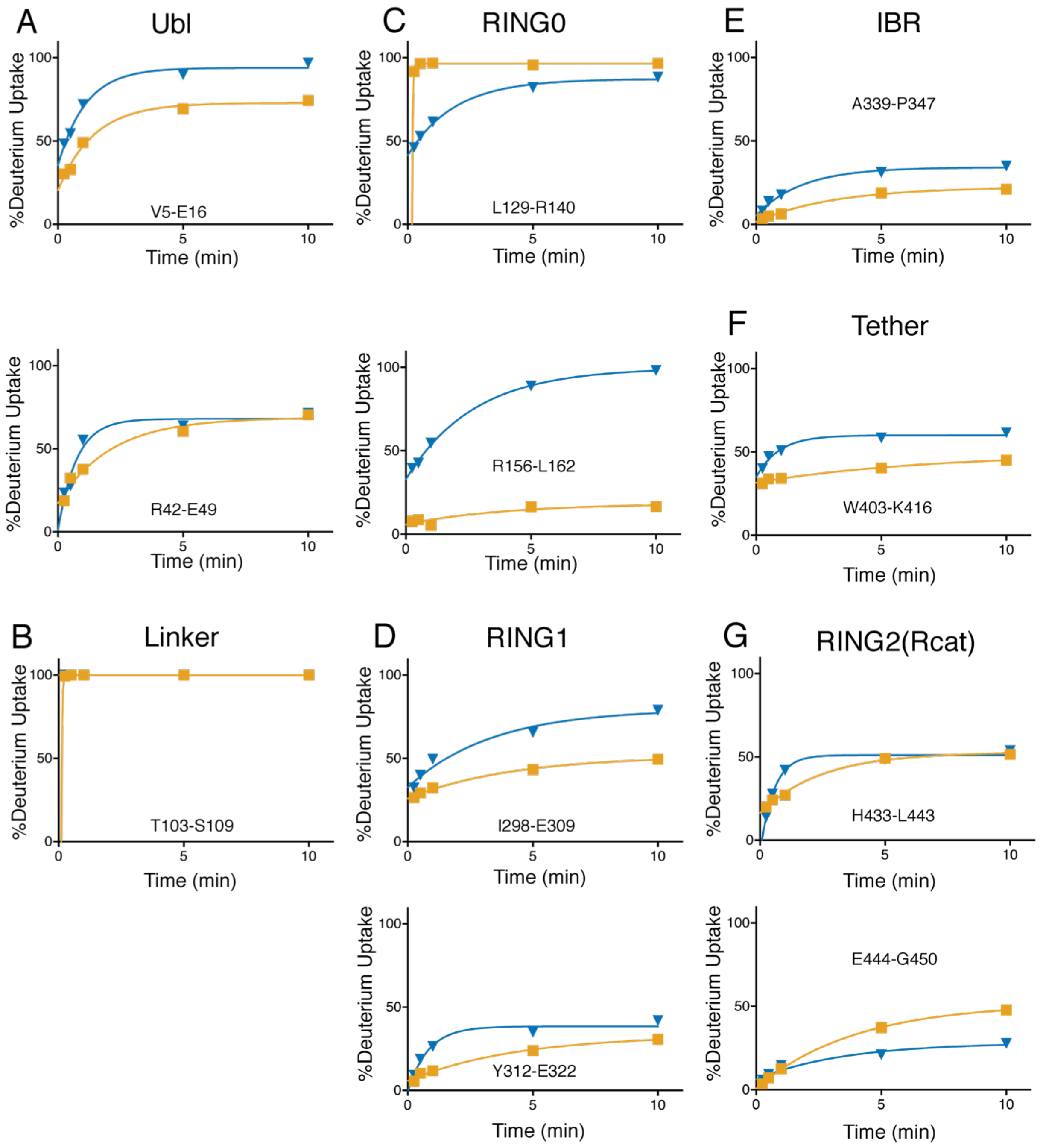

Figure S6. Selected deuterium uptake curves measured from HDX experiments using mass spectrometry. The curves show the % deuterium incorporation for a given peptide as a function of time (minutes) for pParkin:pUb (■) and pParkin:pUb in complex with UbcH7-Ub (▼) in (A) Ubl/pUbl, (B) linker, (C) RING0, (D) RING1, (E) IBR, (F) tether and (G) RING2/Rcat domains.

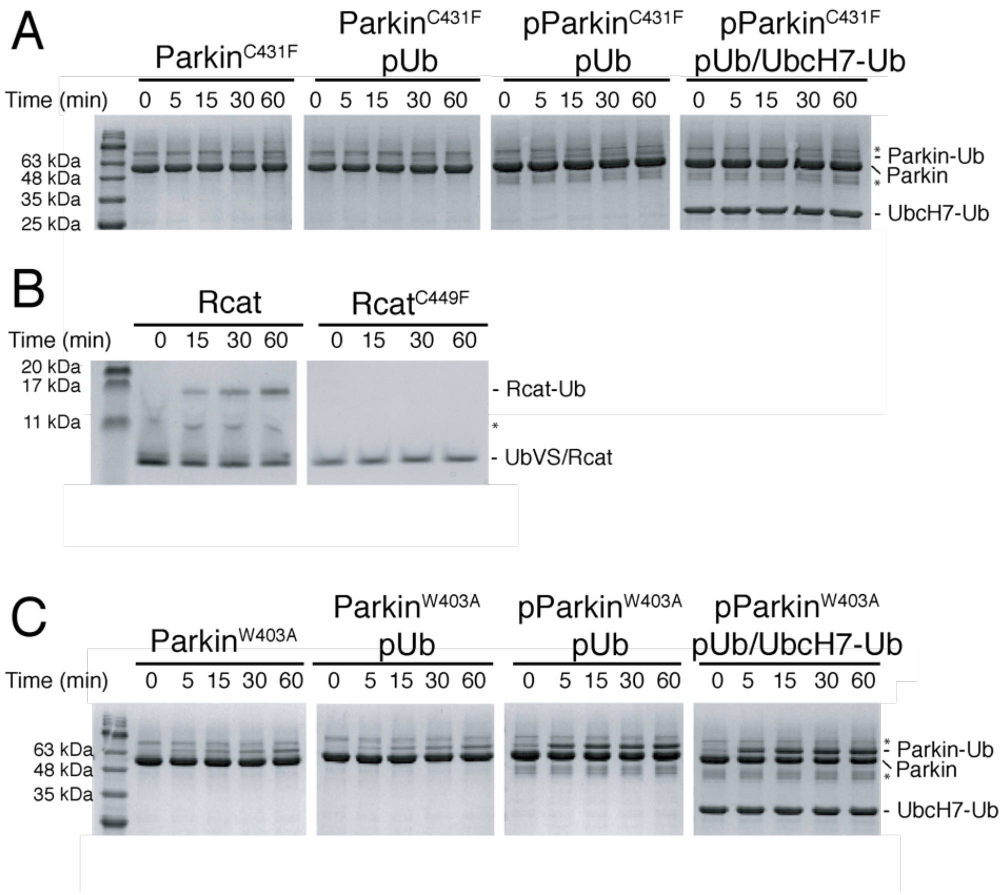

Figure S7. Reaction of the catalytic C431 residue in parkin, parkin^{W403A} and the RING2(Rcat) with a UbVS probe. (A) Experiments utilizing the ARJP substituted parkin^{C431F} show no formation of a UbVS adduct. (B) Reactivity of RING2(Rcat) or Rcat^{C431F} show reactivity is dependent of C431 in the RING2(Rcat) domain only. (C) Experiments with parkin^{W403A} show some reactivity of C431 is noted in the absence of phosphorylation or inclusion of pUb. All samples were taken at the times indicated (0-60 minutes) following addition of UbVS and visualized by SDS-PAGE.
