# Supplementary material for: Synergistic recruitment of UbcH7~Ub and phosphorylated Ubl domain triggers parkin activation
Source: EMBO J. 2018 Nov 16;37(23):e100014. doi: 10.15252/embj.2018100014 (PMC6276879; doi:10.15252/embj.2018100014)
Supplement: Supplementary file 2 — Expanded View Figures PDF [file EMBJ-37-e100014-s002.pdf]

Expanded View Figures

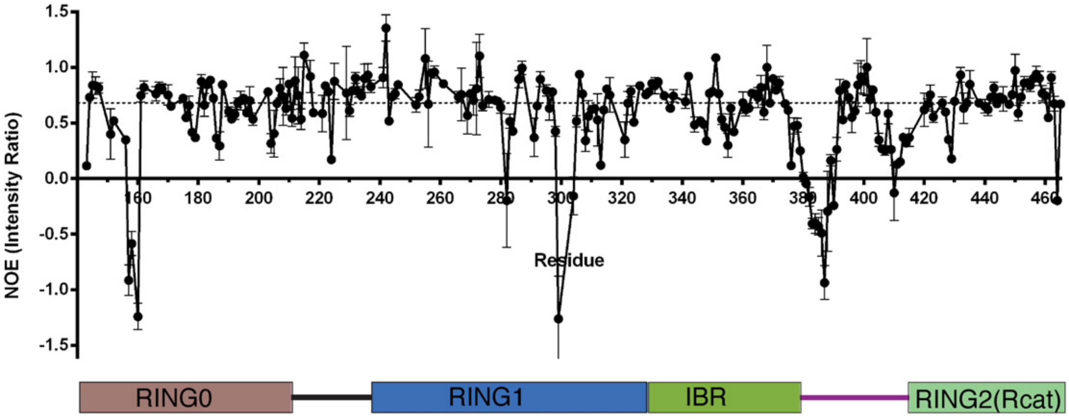

Figure EV1. Backbone flexibility of R0RBR parkin in the R0RBR:pUb complex.

The nOe intensity ratio measured from ^{1}H–^{15}N heteronuclear nOe experiments is plotted per residue. A schematic of R0RBR parkin is shown below the data. The average nOe for the protein is shown (dotted line), and nOes below this indicate increased flexibility. Experiments were measured at 600 MHz using ^{2}H,^{13}C,^{15}N-labelled R0RBR parkin bound to pUb in 25 mM HEPES, 100 mM NaCl and 500 µM TCEP (pH 7.0) at 25°C. Saturation and non-saturation experiments were done in duplicate and averaged. Error bars denote the ± standard deviation from duplicate pairs of experiments.

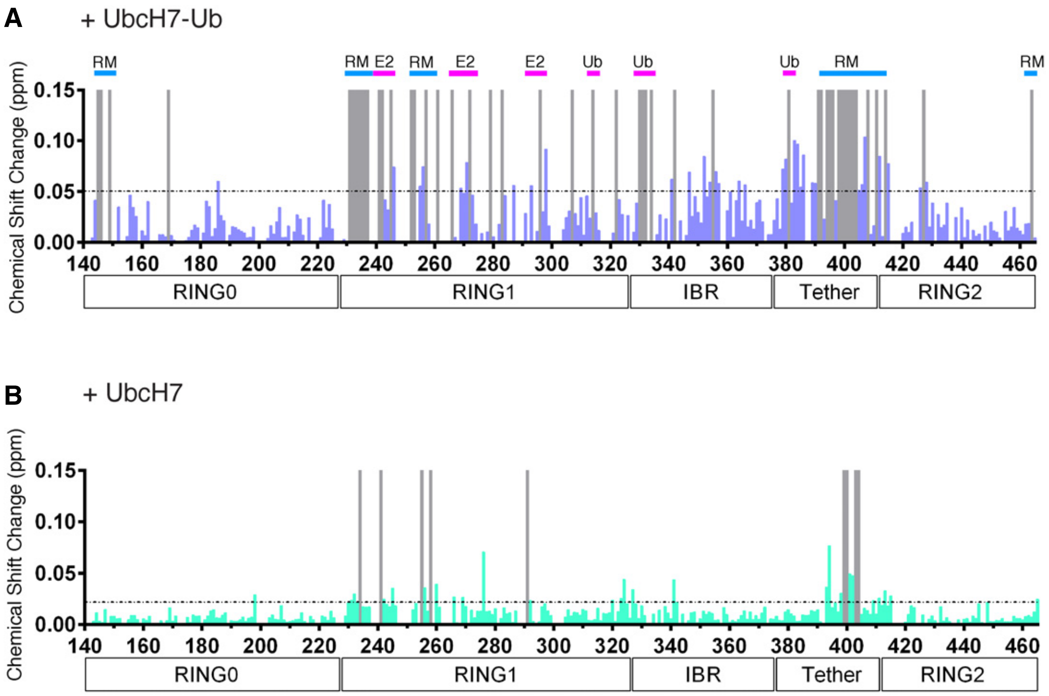

Figure EV2. Chemical shift changes used to map the UbcH7 and UbcH7-Ub binding sites with the R0RBR:pUb complex.

A, B Chemical shift changes were measured in ^{1}H–^{15}N TROSY NMR spectra of ^{2}H,^{13}C,^{15}N-labelled R0RBR parkin in complex with ^{2}H-labelled pUb upon addition of (A) ^{2}H-labelled UbcH7-Ub or (B) ^{2}H-labelled UbcH7. Grey bars indicate that the resonance was undetectable in spectra with UbcH7-Ub or UbcH7 added due to a large change in position and/or broadening. Horizontal dashed lines indicate the average chemical shift change + one standard deviation. In (A), regions are identified either as UbcH7 (E2) and Ub binding (magenta bars) or as a result from re-modelling of the RING0/RING1/RING2 interface (RM, blue bars). Data were collected at 25°C in a buffer containing 25 mM HEPES, 50 mM NaCl and 500 µM TCEP (pH 7.0).

© 2018 The Authors

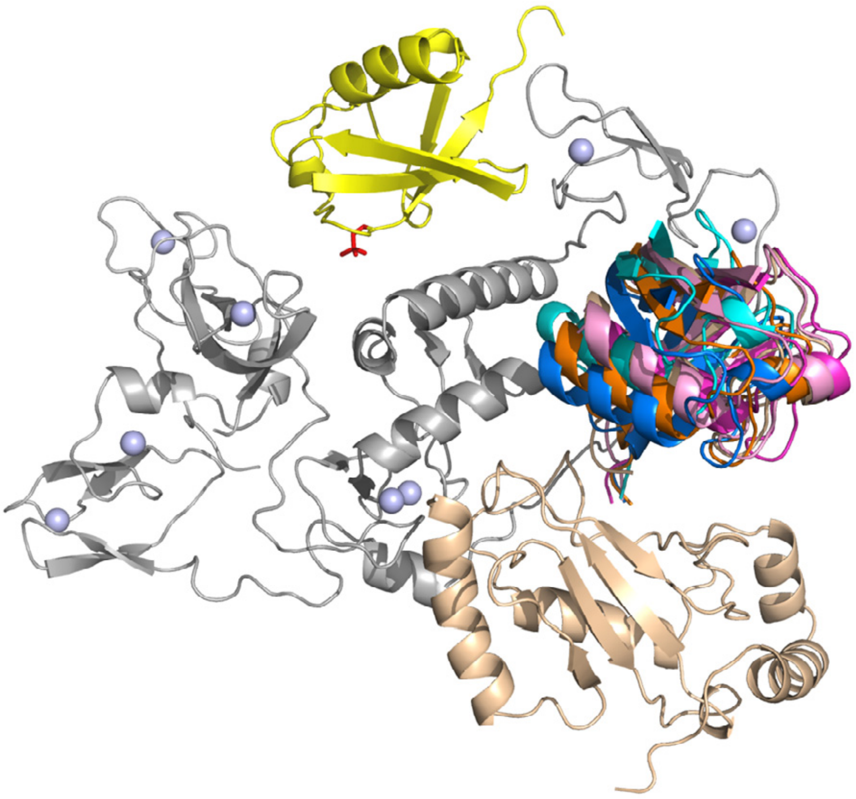

Figure EV3. HADDOCK structures show variable Ub orientations in UbcH7-Ub bound to R0RBR: pUb.

The Ub position in the E2-Ub conjugate is shown for a selection of five HADDOCK models along with the lowest energy model (cyan). The superposition was prepared by superimposing the parkin (grey) and UbcH7 (wheat) portions of the models which show excellent agreement. The orientation of the complex is similar to that for Fig 2C. In the figure, only a single parkin, UbcH7 and pUb (yellow) molecule is shown for clarity.

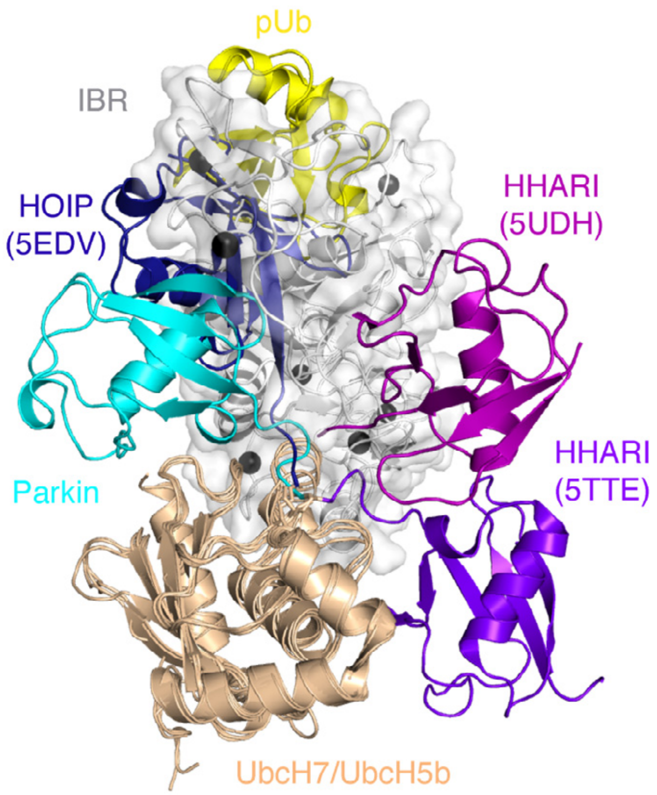

Figure EV4. Comparison of E2-Ub conjugate conformations when bound to different RBR E3 ligases.

The structure of the UbcH7-Ub conjugate (cyan Ub) bound to R0RBR parkin (grey surface) in complex with pUb (yellow ribbon) is shown as determined from HADDOCK calculations in this work. Cartoon diagrams of the E2-Ub conjugates in complex with HOIP (UbcH5b-Ub, navy Ub; PDB 5EDV), HHARI (UbcH7-Ub, magenta Ub; PDB 5UDH) and HHARI (UbcH7-Ub, purple Ub; PDB 5TTE) are shown. The relative arrangement of the Ub molecules within the E2-Ub conjugates was created by superimposing the backbone atoms for the E2 proteins (beige).

© 2018 The Authors
